# Efficacy and Safety of High Vs Standard Daptomycin Doses Examined in Chinese Patients With Severe Burn Injuries by Pharmacokinetic Evaluation

**DOI:** 10.1093/jbcr/iraa020

**Published:** 2020-02-01

**Authors:** Yingzi Huang, Guozhong Lv, Linlin Hu, Yunfu Wu, Nan Guo, Yugang Zhu, Lingtao Ding, Qing Li, Songqiao Liu, Yi Yang, Hua Shao

**Affiliations:** 1 Department of Critical Care Medicine, Zhongda Hospital, School of Medicine, Southeast University, Nanjing, China; 2 Department of Burn and Plastic Surgery, Wuxi Third People’s Hospital, China; 3 Department of Pharmacy, Zhongda Hospital, School of Medicine, Southeast University, Nanjing, China; 4 Department of Critical Care Medicine, Suzhou Municipal Hospital, China; 5 School of Pharmacy, China Pharmaceutical University, Nanjing, China

## Abstract

Previous studies and the concentration-dependent antibacterial actions of daptomycin suggested that a high dose would be needed for difficult-to-treat infections in burn patients. Here, we evaluated the effects of administration of low and high doses of daptomycin in patients with severe burn injuries. The study retrospectively analyzed 10 patients with severe burn injuries, using pharmacokinetic (PK) and pharmacodynamic (PD) evaluations of daptomycin doses given to combat serious infections. Daptomycin was administered as a single dose or by multiple doses intravenously at a standard dose of 6 mg/kg/d or a high dose of 12 mg/kg/d for 7 to 14 days. The serum concentrations of daptomycin from patients were analyzed by liquid chromatography–mass spectrometry/mass spectrometry (LC-MS/MS). Burn injury patients treated with high-dose daptomycin had a linear PK profile and a negative correlation between the AUC_0–24_ and Baux score (*R*^2^ = .953 and *R*^2^ = .801). The *C*_max_, AUC_0–24_, and *t*(h)_½_ increased significantly compared with patients given a standard dose. The efficacy of daptomycin against *Staphylococcus aureus* showed significantly higher rates of (AUC_0–24_)/MIC and *C*_max_/MIC after high-dose daptomycin compared with the standard dose, reflected in a significant correlation between a high dose and the Baux score (*r* = .976, *P* < .001). Positive *S. aureus* cultures from two of three high-dose and none of two daptomycin low-dose patients converted from positive to negative after therapy. No serious adverse events or discontinuation of the drug occurred during the treatment period. Daptomycin doses up to 12 mg/kg/d were well tolerated in Chinese patients with severe burn injuries, which were complicated by infections with *S. aureus*.

## BACKGROUND

Patients with severe thermal injuries are vulnerable to infections for various reasons, such as trauma, mechanical ventilation, dermatoplasty, and blood transfusion.^1^ Methicillin-resistant *Staphylococcus aureus* (MRSA) is one of the most common pathogens involved in severe and complicated skin and soft tissue infections, which might result in MRSA bacteremia with increased mortality in burn injury wards.^2–4^ Daptomycin is used to treat refractory infections caused by MRSA due to its distinct mechanism of action.^5,6^ Daptomycin is a cyclic lipopeptide antibiotic derived from fermentation of *Streptomyces roseosporus*, with good in vitro activity against *Staphylococci* (including *MRSA*), *Enterococci* (including glycopeptide-resistant *Enterococci* [GRE]), *Streptococci*, *Corynebacteria*, and stationary-phase *Borrelia burgdorferi* persisters. First discovered in the late 1980s, daptomycin is administered intravenously once daily at a dose of 4 to 6 mg/kg/d for the treatment of complicated skin and skin structure infections or for *S. aureus* bacteremia and *S. aureus* right-sided endocarditis, respectively.^7–9^ Although high doses of daptomycin are associated with adverse effects on skeletal muscle and life-threatening eosinophilic pneumonia,^10–13^ it has been demonstrated that it exerts bactericidal activity in a concentration-dependent manner^14^ and is effective and well tolerated at higher doses (>6 mg/kg/d) for difficult-to-treat Gram-positive infections.^15^ In addition, the concentration of plasma daptomycin in patients with severe burn injuries significantly decreased,^13^ suggesting that a high dose of daptomycin should be considered to achieve the desired therapeutic concentration in these patients as a standard dose would in a normal population.

However, there are no clinical trial data on the use of daptomycin at doses >6 mg/kg/d for the treatment of patients with severe burn injuries in China. In the present study, we analyzed the pharmacokinetic and pharmacodynamic properties of high-dose daptomycin and compared the results with the standard dose given to patients with severe burn injuries resulting from one serious accident. In addition, the safety and tolerability of high-dose daptomycin was investigated.

## METHODS

### Data Source

This study was a single-center retrospective study of Chinese patients with one serious accident-caused burn injury. The therapies were conducted at the Burn Intensive Care Unit of Wuxi Third People’s Hospital from August 2014 to October 2014. The medical record database was established simultaneously to record, save, and process all treatment data, which included the demographic information of the burn injury patients, the use and dosage of antimicrobial agents, as well as the efficacy of high daptomycin doses with PK/PD analyses, which was applied according to a previously published article.^13^ The database was maintained by the hospital medical records management department of Wuxi Third People’s Hospital, from which the permission to use de-identified data for assessment and publication was obtained. The study is approved by the ethics committee of the Zhongda Hospital (no.: 2019ZDSYLL165-P01).

### Basic Characteristics of Enrolled Patients With Severe Burn Injuries

The severe burn injury patients (age range 20–50 years), were at least 7 days postburn injury and received either standard-dose (6 mg/kg/d) or high-dose (12 mg/kg/d) daptomycin. In this study, severe burn injury was defined as >50% body surface area burns. Inclusion criteria were completion of initial fluid resuscitation, and confirmed or suspected Gram-positive bacterial infection in the blood stream, skin, and/or soft tissues. Exclusion criteria were allergy to daptomycin; confirmed or suspected infections caused by Gram-negative bacteria; undergoing renal replacement therapy; pregnancy or breast-feeding; a history of rhabdomyolysis; or elevated creatine phosphokinase. Baux scores for predicting burn-caused mortalities were evaluated as: Burn area + age + 17 × (inhalation injury, yes = 1, no = 0).

### Medication

Patients received a single 30-minute intravenous infusion of 6 mg/kg/d or 12 mg/kg/d of daptomycin (Cubicin, AstraZeneca) based on the patient’s actual body weight through an infusion pump. High doses were applied, when low doses did not lead to standard dose serum concentrations of daptomycin. Plasma samples were routinely obtained at 0, 0.25, 0.5, 1.0, 1.5, 2.5, 4.5, 6.5, 8.5, 10.5, 12.5, and 24.5 hours after the start of the infusion. Multiple-dose medication treatment means that daptomycin was administered to burn injury patients as one injection every day of either the standard dose or a high dose. Subsequently, the plasma concentration of daptomycin was measured and pharmacokinetic parameters determined in the blood at 0, 0.25, 0.5, 1.0, 1.5, 2.5, 4.5, 6.5, 8.5, 10.5, 12.5, and 24.5 hours on day 6, which is a routine procedure in the Burn Intensive Care Unit department. During intravenous infusion, vigorous agitation or shaking during drug dissolution was not permitted, thus preventing the formation of gas bubbles.

### Blood Sample Collection and Measurements

Blood samples for plasma assays were collected into EDTA anticoagulant tubes at the following time points: 0 (the beginning of the infusion), 0.25, 0.5, 1.0, 1.5, 2.5, 4.5, 6.5, 8.5, 10.5, 12.5, and 24.5 hours on days 1 and 6 (which served as data for a single dose); and at 0, 0.5, 4.5, 12.5, and 24.5 hours on days 2 to 5 (which served as data for multiple-dose administrations). The samples were centrifuged and stored at −80°C until required for analysis.

PK evaluation was performed by measuring the daptomycin levels in blood samples, using liquid chromatography–mass spectrometry/mass spectrometry (LC-MS/MS) (Waters Micromass Quattro Micro API LC-MS/MS). Chromatographic separation was performed at 35°C using a RP-C18 (Welch Ultimate, 3.0 mm × 150 mm) analytical column. Letrozole (200 g/ml) (National Institutes for Food and Drug Control) was added to the plasma after protein precipitation by acetonitrile, and was used as the internal standard. The mobile phase consisted of 0.1% acetic acid (phase A) and acetonitrile (phase B) using gradient elution at a flow rate of 0.32 ml/min. The grade was 50:50 (phase A:phase B) at time zero, maintained for 5.1 minutes, changed to 0:100 over 0.2 minutes, kept for 3.7 minutes, and then changed to the initial condition in 0.3 minute, giving a total run time of 16 minutes. Quantification was performed in multiple-reaction monitoring (MRM) mode, with specific transitions of protonated precursor ions to produce ions at *m/z* 811.45 to *m/z* 159.25 [M+H]^+^ for daptomycin and *m/z* 286.42 to *m/z* 217.29 [M+H]^+^ for IS. Nitrogen was used as the nebulizer and drying gas, and the gas temperature and flow were adjusted to 350°C and 10 l/min, respectively. The fragment energy and the optimized collision energy was 35 eV/30 eV (IS) and 25 eV/15 eV (IS), respectively.

#### Pharmacokinetic and Pharmacodynamic Parameter Analysis.

PK parameters were calculated using DAS 2.0. The area under the concentration–time curve between 0 and 24 h (AUC_0–24_) and 0 to infinity (AUC_0–∞_) was calculated using the linear trapezoidal rule. The maximum plasma concentration (*C*_max_) and the time at which it occurred (*T*_max_) were determined directly from the measured plasma concentration–time without interpolation. The minimum plasma concentration (*C*_min_) was calculated from the measured daptomycin concentration immediately before dosing. The elimination rate constant (K_e_) was calculated from the terminal portion of the concentration–time curve and the elimination half-life (*t*_½_) was calculated as the ratio *t*_½_ = 0.693/K_e_. The total body clearance (CL) was obtained from dose/AUC_0–∞_. The volume of distribution (*V*_d_) was calculated using the equation CL/K_e_.

The pharmacodynamics parameters of daptomycin in this article are discussed only for *S. aureus*. The minimum inhibitory concentration (MIC) value of daptomycin was examined according to the guidelines of the Clinical and Laboratory Standards Institute (CLSI), which gives the lowest concentration of daptomycin with bactericidal activity against *S. aureus*.

### Statistical Analysis

A paired t test was used to compare hepatorenal function alterations before and 7 days after daptomycin therapy. Analysis of variance was used to determine the PK parameters of standard-dose or high-dose daptomycin after single or multiple administrations in patients with severe burns. Correlation analysis was assessed between the Baux score and the AUC_0–24_ steady state of daptomycin. All statistical analyses were conducted using SAS^™^ 9.3 software (Cary, NC) and a P value <.05 was considered to be statistically significant.

## RESULTS

### Basic Demographics of Patients With Severe Burn Injuries

A total of 10 patients with severe burns aged from 20 to 50 years were enrolled in the study. The cohort of patients comprised six men (6/10) and four women (4/10). The TBSA of the patients had a mean of 95.1 ± 15.2% (range 50–99.0%). MRSA infections were identified in five patients (5/10) and daptomycin was injected into three and seven burn patients at doses of 6 or 12 mg/kg/d, respectively (Table 1). Table 2 shows that the baseline information of patient characteristics were not significant different between the low- and high-dose daptomycin groups before onset of therapy.

**Table 1.** Demographics of patients

Patient No.	Age Range (yr)	Sex	Weight (kg)	Height (cm)	Days After Burn	TBSA (%)	Full Thickness Burns Area (%)	Baux Score	Culture Site	Organism Isolated	Survivor 90 d
High-dose group (12 mg/kg/d)											
1	30–40	M	70	167	80	99	95	156			Yes
2	20–30	M	67	170	47	95	90	140	Blood cultures/wound	MRSA	Yes
3	20–30	F	55	162	72	98	85	145			No
4	20–30	M	65	169	49	98	64	135	Blood cultures/wound	MRSA	Yes
5	40–50	F	65	155	33	50	40	110			Yes
6	40–50	M	68	167	19	85	75	155	Blood cultures	MRSA	Yes
7	40–50	F	53	156	25	95	90	162			No
Standard-dose group (6 mg/kg/d)											
8	40–50	M	72	167	60	99	95	158	Wound	MRSA	Yes
9	20–30	F	55	162	63	98	85	145			No
10	40–50	M	60	158	43	95	80	156	Wound	MRSA	Yes

*MRSA*, methicillin-resistant *Staphylococcus aureus*; Baux score = Burns area + Age + 17 × (inhalation injury, yes = 1, no = 0).

**Table 2.** Comparison of basic patient data before onset of therapy

Characteristics	High-Dose Group (12 mg/kg/d) *n* = 7	Standard-Dose Group (6 mg/kg/d) *n* = 3	*P*
Age	36.28 ± 10.53	38.67 ± 7.57	.735
Sex (F/M)	3/4	1/2	1.000
% TBSA	88.6 ± 17.7	97.3 ± 2.1	.436
% Full thickness burns	77.0 ± 19.4	86.7 ± 7.6	.438
Inhalation burn yes/no	3/4	1/2	1.000
Burn wound excision			
Partial	7 (100%)	3 (100%)	1.000

### Comparison of Hepatorenal Function Alteration Before and After Treatment in the Two Dosage Groups

We compared the hepatorenal function index before and on day 7 after administration of daptomycin (Table 3). No significant differences in baselines of endogenous creatinine clearance rate (Ccr), blood urea nitrogen (BUN), total protein (TP), and albumin concentrations were found in patients before standard- and high-dose daptomycin treatment. After high-dose daptomycin therapy, no differences were found in the Ccr, BUN, and TP values before and after therapy. However, the mean concentration of albumin on day 7 after high-dose daptomycin treatment was significantly higher than the baseline value (40.5 ± 4.6 vs 35.0 ± 6.5, respectively, *P* = .006) (Table 3).

**Table 3.** Hepatorenal function index of patients before and after medication

Patient No.	Before Daptomycin Treatment				After Daptomycin Treatment (7 days)			
	Ccr (ml/min)	BUN (mmol/l)	TP (g/l)	Alb (g/l)	Ccr (ml/min)	BUN (mmol/l)	TP (g/l)	Alb (g/l)
High-dose group (12 mg/kg/d)								
1	213.52	11.1	56.0	37.0	208.21	10.94	63.8	39.5
2	223.28	6.4	70.0	43.0	222.66	6.54	75.2	47.1
3	107.6	44.2	72.0	24.0	70.23	54.12	67.00	36.00
4	222.86	12.9	50.0	30.0	240.70	8.75	60.00	38.00
5	207.44	7.4	60.0	38.0	178.52	6.35	68.2	42.9
6	210.86	8.9	67.0	40.0	212.61	7.82	76.4	44.9
7	119.13	10.7	53.0	33.0	90.47	14.6	52.6	35.1
Average ± SD	186.4 ± 50.3	14.5 ± 13.3	61.1 ± 8.6	35.0 ± 6.5	174.8 ± 67.4	15.6 ± 17.2	66.2 ± 8.4	40.5 ± 4.6
*P* (before vs after)					.180	.557	.057	**.006**
Standard-dose group (6 mg/kg/d)								
8	209.4	12.5	60.0	34.0	169.0	20.1	66.0	32.0
9	164.0	15.8	70.5	29.0	112.0	16.3	64.0	32.0
10	211.4	10.1	49.0	33.7	220.0	13.9	56.0	35.0
Average ± SD	194.9 ± 26.8	12.8 ± 2.9	59.8 ± 10.8	32.2 ± 2.8	167.0 ± 54.0	16.8 ± 3.1	62.0 ± 5.3	33.0 ± 1.7
*P* (before vs after)					.468	.178	.767	.694

*Ccr*, endogenous creatinine clearance rate; *BUN*, blood urea nitrogen; *TP*, total protein; *Alb*, albumin.

### Comparison of PK Parameters After Standard-Dose and High-Dose Daptomycin Following Single or Multiple Administrations in Patients With Severe Burns

Treatment with multiple high doses of daptomycin induced a significant increase in *C*_max_, AUC_0–24_, and *t*(h)_½_ compared with single high-dose daptomycin administration (79.17 ± 16.84 vs 70.90 ± 16.35, *P* = .012; 641.51 ± 177.91 vs 456.8 ± 138.9, *P* = .002; and 8.10 ± 1.91 vs 7.40 ± 2.50, * P* < .000, respectively) (Table 4). However, the CL decreased significantly in multiple high-dose daptomycin-treated patients compared with single high-dose treatment (19.80 ± 4.78 vs 22.5 ± 4.1, *P* = .040). In contrast, no significant differences in PK parameters were found between single and multiple administrations in patients treated with 6 mg/kg/d of daptomycin.

**Table 4.** Comparison of pharmacokinetic parameters of daptomycin treatment between single and multiple administrations in patients with severe burn injuries

Patients No.	Single Dose					Multiple Dose				
	*C*_max_ (µg·h/ml)	AUC_0–24_ (µg·h/ml)	*t*(h)_½_	*V*_d_ (l/kg)	CL (ml/h/kg)	*C*_max_ (µg·h/ml)	AUC_0–24_ (µg·h/ml)	*t*(h)_½_	*V*_d_ (l/kg)	CL (ml/h/kg)
High-dose group (12 mg/kg/d)										
1	69.08	486.9	0.5	0.26	21.9	82.18	588.2	8.8	0.26	20.40
2	66.54	511.2	0.5	0.21	21.6	67.86	679.0	8.5	0.22	17.67
3	43.51	426.7	0.5	0.25	25.4	55.22	580.7	6.7	0.20	20.66
4	80.29	477.6	0.5	0.29	23.3	79.14	709.6	6.6	0.16	16.91
5	97.99	809.9	1.5	0.10	14.1	110.45	987.6	5.7	0.10	12.15
6	70.99	439.4	0.5	0.15	26.7	77.61	489.1	9.1	0.32	24.53
7	67.92	399.0	0.5	0.41	24.3	81.69	456.2	11.3	0.43	26.30
Average ± SD	70.90 ± 16.35	456.8 ± 138.9	7.40 ± 2.5	0.24 ± 0.1	22.5 ± 4.1	79.17 ± 16.84	641.5 ± 177.9	8.10 ± 1.91	0.24 ± 0.11	19.80 ± 4.78
*P* (single vs multiple)						**.012**	**.002**	**<.000**	.936	**.040**
Standard-dose group (6 mg/kg/d)										
8	29.26	273.7	4.97	0.14	21.92	32.27	394.7	6.093	0.15	17.57
9	24.09	197.0	6.737	0.27	30.45	30.97	249.5	7.958	0.44	38.32
10	26.63	295.2	5.377	0.15	20.32	27.69	330.3	5.349	0.14	18.16
Average ± SD	26.66 ± 2.58	255.3 ± 51.61	5.64 ± 1.0	0.19 ± 0.1	24.2 ± 5.4	30.31 ± 2.36	324.85 ± 72.76	6.47 ± 1.34	0.24 ± 0.17	24.7 ± 11.8
*P* (single vs multiple)						.166	.118	.194	.425	.915
*P* (single vs multiple for all patients)						**.004**	**.000**	**.001**	.525	.211
*P* (high dose vs low dose)	.000	.018	<.000	.448	.586	.000	.020	.221	.983	.356

*C*_max_, maximum plasma concentration; AUC_0–24_, area under the concentration vs time curve from 0 to 24 h; *t*(h)_½_, plasma half-life; *V*_d_, volume of distribution; CL, total body clearance.

Next, we compared the PK parameters of single and multiple administrations of daptomycin, independent of the dose received. Patients with severe burn injuries treated with multiple doses of daptomycin showed a significantly increased *C*_max_, AUC_0–24_ and *t*(h)_½_ compared with patients who received a single administration (79.17 ± 16.84 vs 70.90 ± 16.35, *P* = .004; 641.51 ± 177.91 vs 456.8 ± 138.9, *P* = .000; and 8.10 ± 1.91 vs 7.40 ± 2.50, *P* = .001, respectively), while no significant difference was found in *V*_d_ and CL between single and multiple infusions. Furthermore, we analyzed the PK parameters of single and multiple administrations of standard- and high-dose daptomycin, respectively. While no significant difference was found for *V*_d_ and CL after a single infusion between both doses of daptomycin, *C*_max_, AUC_0–24_ and *t*(h)_½_ in high-dose daptomycin-treated patients increased significantly compared with patients who received 6 mg/kg/d of daptomycin (70.90 ± 16.35 vs 26.66 ± 2.58, *P* = .000; 456.8 ± 138.9 vs 255.3 ± 51.61, *P* = .018; and 7.40 ± 2.50 vs 5.64 ± 1.00, *P* < .000, respectively). As for multiple infusions of daptomycin, patients treated with high-dose daptomycin exhibited a significantly increased *C*_max_ and AUC_0–24_, but *t*(h)_½_, *V*_d_ and CL did not significantly differ in patients treated with 6 mg/kg/d of daptomycin (Table 4).

**Figure 1.**
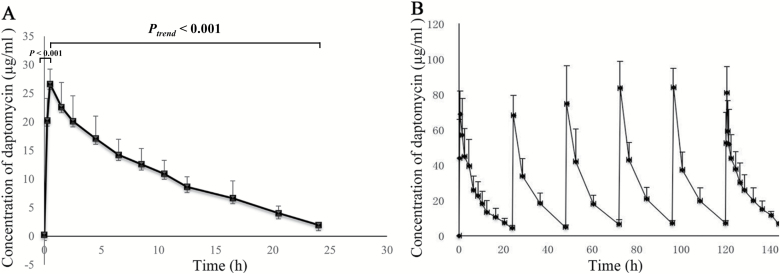
A. Comparison of the concentration–time curve of daptomycin after single administration for three severely burned patients who were given daptomycin at 6 mg/kg/d in 24 hours. B. Comparison of the concentration–time curve of daptomycin at 12 mg/kg/d after single and multiple administration in the seven severely burned patients from days 1 to 6.

**Figure 2.**
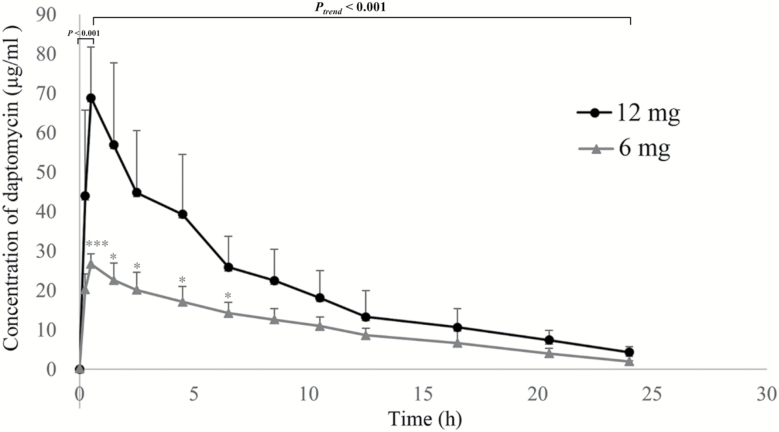
Comparison of daptomycin concentration in severely burned patients treated with a high dose and a standard dose of daptomycin (single administration in 24 hours). ***P < .001, *P < .05, comparing high- and low-dose concentrations at the indicated time points.

**Figure 3.**
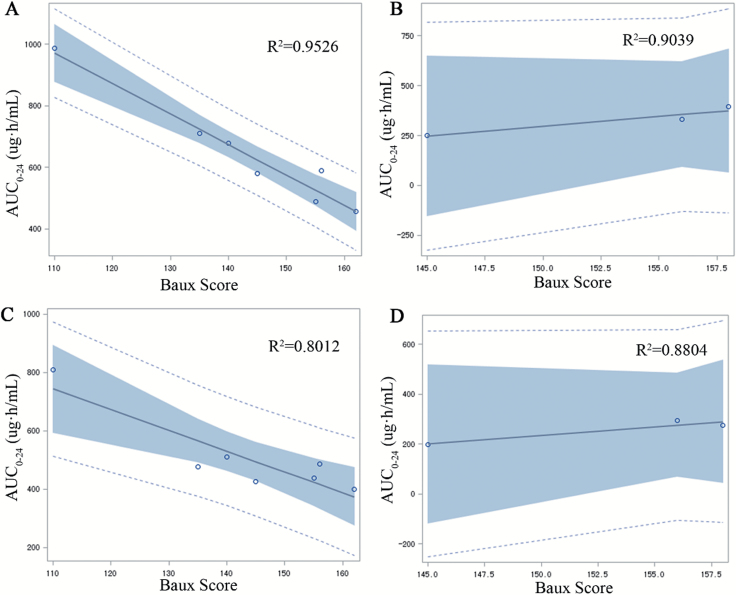
The correlation between the Baux score and steady-state AUC_0–24_ (µg·h/ml) of daptomycin in seven severely burned patients given 12 mg/kg/d and in three patients given 6 mg/kg/d. A. Multiple 12 mg/kg/d administrations. B. Multiple 6 mg/kg/d administrations. C. Single 12 mg/kg/d administration. D. Single 6 mg/kg/d administration. AUC_0–24_, area under the concentration vs time curve from 0 to 24 hours.

Figure 1 shows the concentration–time curve in three patients who received the single 6 mg daptomycin/kg dose (Figure 1A) and the seven patients who received single and multiple doses of 12 mg/kg/d of daptomycin (Figure 1B). The concentration of daptomycin, administered as a single standard dose, reached a peak after about 2 hours from the start of the infusion (*P* < .001) and then gradually decreased to a steady state level after approximately 24 hours (*P* < 0.001) (Figure 1A). Similarly, the plasma concentration of daptomycin increased immediately after the subsequent infusion followed by a decrease to a steady state level after about 24 hours, with the peak being highest after the fourth administration (Figure 1B). Furthermore, the concentration of daptomycin was significantly higher in patients treated with high-dose daptomycin than those treated with 6 mg/kg/d of daptomycin within the first 7 hours after a single administration (*P* < .05), while no significant difference in the daptomycin concentration was found between the two doses after 7 hours (Figure 2). The correlation coefficient between the AUC_0–24_ and Baux score in patients who received a high dose of daptomycin with multiple administration was significantly decreased compared with patients who received a single dose (*R* = −.976 vs −.895, *P* < .05), suggesting a negative correlation between the AUC_0–24_ and Baux score according to the degree of correlation fitting values (*R*^2^ = .953 and *R*^2^ = .801, respectively). In contrast, there was no significant correlation between the AUC_0–24_ and Baux score in patients treated with 6 mg/kg/d of daptomycin between multiple (*P* = .201) and single administrations (*P* = .225), (*R*^2^ = .904 and *R*^2^ = .880, respectively) (Figure 3 and Table 5).

**Table 5.** Correlation between multiple or single infusion of AUC_0–24_ (µg·h/ml) with the Baux score

AUC_0–24_ (µg·h/ml) A2	Baux			
	Multi-12 mg/kg/d	Multi-6 mg/kg/d	Single-12 mg/kg/d	Single-6 mg/kg/d
*R*	.976	.951	−.895	.938
*P*	.000	.201	.007	.225

AUC_0–24_, area under the concentration vs time curve from 0 to 24 h.

Finally, we examined PD parameters for *S. aureus* in severely burned patients with MIC values of 0.5 or 1, guided by CLSI. As given in Table 6, the AUC_0–24_/MIC and *C*_max_/MIC values in patients treated with high-dose daptomycin (12 mg/kg/d) significantly increased compared with 6 mg/kg/d of daptomycin (1283.0 ± 355.8 vs 510.6 ± 103.22, *P* = .007, MIC = 0.5 and 641.5 ± 177.9 vs 255.3 ± 51.61, *P* = .007, MIC = 1; 158.3 ± 33.6 vs 53.32 ± 5.16, *P* = .001, MIC = 0.5; 79.17 ± 16.84 vs 26.66 ± 2.58, *P* = .001, MIC = 1, respectively), but no significant difference in creatine phosphokinase (CPK) levels were found between patients who received a standard dose (6 mg/kg/d) or high dose (12 mg/kg/d).

**Table 6.** The pharmacodynamics parameters of daptomycin at 12 and 6 mg/kg/d for *Staphylococcus aureus* (average ± SD)

Parameters	12 mg/kg/d		6 mg/kg/d		*P*	
	MIC = 0.5	MIC = 1	MIC = 0.5	MIC = 1	MIC = 0.5	MIC = 1
AUC_0–24_/MIC	1283.0 ± 355.8	641.5 ± 177.9	510.6 ± 103.22	255.3 ± 51.61	.007	.007
*C*_max_ (µg/ml)MIC	158.3 ± 33.6	79.17 ± 16.84	53.32 ± 5.16	26.66 ± 2.58	.001	.001

*C*_max_, maximum plasma concentration; AUC_0–24_, area under the concentration *vs* time curve from 0 to 24 h; *t*(h)_½_, plasma half-life; *MIC*, minimum inhibitory concentration.

*Staphylococcus aureus* cultures from two of three patients treated at 12 mg/kg/d and none from two patients in the 6 mg/kg group converted from positive to negative after therapy (Table 7).

**Table 7.** The changes of bacterial isolates from 10 patients with infected burn wounds after daptomcin therapy

Staphylococcus aureus methicillin-resistance patterns	Before Daptomcin Therapy		After Daptomcin Therapy	
	High Dose (12 mg/kg/d)	Standard Dose (6 mg/kg/d)	High Dose (12 mg/kg/d)	Standard Dose (6 mg/kg/d)
Methicillin-resistant *Staphylococcus aureus* positive cultures, *n*	3	2	1	2
Changes of methicillin-resistant *S. aureus* cultures from positive to negative, *n* (%)			2/3 (66.7)	0/2 (0.0)

### High-Dose Daptomycin Is a Useful Drug to Treat Patients With Severe Burn Injuries

Increased CPK is the most common adverse reaction to daptomycin, and there was only one patient (no. 3) that showed a transient increase of CPK on day 3 after receiving high-dose daptomycin. The level returned to a normal value without discontinuation of daptomycin treatment. There were no significant CPK differences in the indicated time points between the groups (Table 8). No serious adverse events (SAEs) and nonserious AEs, which could have led to discontinuation of daptomycin occurred during the study.

**Table 8.** The creatine phosphokinase (CPK, U/L) levels of patients during the treatment period

Patient No.	Baseline	Day 1	Day 3	Day 7
High-dose group (12 mg/kg/d)				
1	8	4	11	10
2	12	13	18	16
3	20	48	197	107
4	8	10	6	7
5	21	34	33	15
6	36	27	26	19
7	24	23	59	15
Average ± SD	18.4 ± 10.1	22.7 ± 15.2	50.0 ± 67.1	27.0 ± 35.5
Standard-dose group (6 mg/kg/d)				
8	10	7	11	9
9	9	16	19	16
10	17	22	25	28
Average ± SD	12.0 ± 4.4	15.0 ± 7.5	18.3 ± 7.0	17.7 ± 9.6
P*	.330	.439	.262	.675

*P values comparing high- and standard-dose groups.

## DISCUSSION

The antibiotic daptomycin exhibits concentration-dependent activity against Gram-positive bacteria and is used to treat *Staphylococcus* bacteremia and right-sided endocarditis at a dose of 6 mg/kg/d.^16^ However, both in vivo and in vitro PK/PD models suggested that a higher daptomycin dose (12 mg/kg/d) might be required to treat these infections.^17,18^ We compared the PK/PD parameters of high- and standard-dose daptomycin after single or multiple administrations in severely burned patients. High-dose daptomycin had a linear PK profile and showed a negative correlation between AUC_0–24_ and the Baux score. In addition, the efficacy of daptomycin against *S. aureus* showed a significantly higher (AUC)/MIC and *C*_max_/MIC ratio after a high dose compared with the standard dose. This finding indicates that for more than 50% TBSA, daptomycin at a dose of 12 mg/kg/d of body weight per day is required to achieve drug exposures similar to healthy volunteers who received 6 mg/kg/d.^13^

In addition, consistent with previous PK studies conducted in healthy volunteers,^19,20^ we demonstrated a dose-dependent increase in *C*_max_ and AUC_0–24_ values. However, the *C*_max_ and AUC_0–24_ values determined in patients with severe burns treated with high-dose daptomycin were about 1/2 to 1/3 lower than in healthy volunteers after single or multiple doses.^20^ In contrast to one phase 1 study on healthy volunteers, when the steady-state concentration of daptomycin peaked at day 4 for all dose levels, our study revealed that the plasma concentration of daptomycin peaked to similar levels after each administration and decreased to similar levels within 24 hours. It is believed that daptomycin binding to plasma proteins is independent of the dose and plasma concentration,^20^ and that the plasma half-life is also independent of the administration method and dose. However, we detected an increased *t*(h)_½_ in patients who received a high dose of daptomycin. Different metabolic processes involving daptomycin and plasma proteins in healthy individuals and patients with burn injuries may explain these discrepancies.

The AUC and days after the burn injury have been reported in previous PK studies with single-dose intravenous daptomycin administration.^13^ We demonstrated that the average AUC_0–24_ value in burn patients treated with single and multiple high-dose daptomycin was negatively correlated with the Baux score, but had no correlation with the number of days from the burn injury. The severe condition of our patients, lack of improvement in their physical condition in the short-term, and different metabolic processes may have contributed to the differences noted in the previous report. Since the PK of daptomycin was linear and dose-proportional,^20^ the equation between the steady-state AUC_0–24_ and Baux score was used to predict the plasma concentration and permitted the dose of daptomycin to be calculated according to the target AUC_0–24_.

*C*_max_/MIC and AUC/MIC ratios are considered the best predictors for the efficacy of daptomycin against infections caused by *S. aureus*.^17^ We found an increased *C*_max_/MIC and AUC_0–24_/MIC with MIC at 0.5 or 1 in patients treated with high-dose daptomycin compared with a standard dose, but no significant difference of CPK in either standard-dose or high-dose treated patients. It is noteworthy that all patients in our study received supplemental plasma albumin and that the serum albumin level was controlled within the normal range. Thus, the increased serum albumin in patients treated with high-dose daptomycin would likely have no relationship with the steady state AUC_0–24_ of high-dose daptomycin.

Daptomycin at 12 mg/kg/d elicited no serious adverse events or treatment discontinuations during the entire study period suggesting a good tolerability of daptomycin dosage up to 12 mg/kg/d administered intravenously once daily for 14 days. Although daptomycin has well known adverse effects, the FDA has identified seven confirmed daptomycin-associated eosinophilic pneumonia cases and confirmed that the victims were all older than 60 years, with symptoms occurring within 2 weeks of initiation of daptomycin treatment.^10,12,21^ There have also been a few case reports that pointed out serious respiratory complications associated with daptomycin therapy.^22,23^ Healthcare professionals should be aware of these life-threatening adverse events when treating burn patients with daptomycin.

Limitations of the present study were its retrospective design and the low sample number which was due to the fact that the patients were all affected by single serious fire disasters which is difficult to expand to a larger sample size.

## CONCLUSIONS

The present study demonstrated that 12 mg/kg/d of daptomycin was well tolerated in patients with severe burn injuries who may have different PK and PD parameters than normal healthy subjects.

## Data Availability

Data are available from the authors upon reasonable request.
